# Effects of ZnFe_2_O_4_ Nanoparticles on Development and Rhythmic Behavior of *Drosophila melanogaster*

**DOI:** 10.3390/toxics13090779

**Published:** 2025-09-14

**Authors:** Wenhao Yan, Yunfan Guo, Penghui Li, Ziyan Zhang, Jinjun Yang, Yongyan Sun

**Affiliations:** 1 School of Environmental Science and Safety Engineering, Tianjin University of Technology, Tianjin 300384, China; 2 Institute of Urban Environment, Chinese Academy of Sciences, Xiamen 361021, China

**Keywords:** *Drosophila melanogaster*, ZnFe_2_O_4_-NPs, rhythmic behavior, development, stress responses

## Abstract

**Objectives**: This study planned to determine the biological effects associated with ZnFe_2_O_4_-NPs exposure using *Drosophila melanogaster* as an in vivo model. **Methods**: ZnFe_2_O_4_-NPs were hydrothermally synthesized, and the development of offspring flies were evaluated via dietary exposure to different doses of ZnFe_2_O_4_-NPs (0, 200, 400, 600 μg/mL). Rhythmic behaviors of parent male flies were monitored. **Results**: Internalization of ZnFe_2_O_4_-NPs through the intestinal barrier occurred. Oral intake of ZnFe_2_O_4_-NPs decreased the eclosed adult numbers and perturbed the insect developmental process. In male flies, significant upregulation of HSPs and Turandot family genes was detected, accompanied by ROS reduction and suppressed antioxidant defense responses, and exposure of ZnFe_2_O_4_-NPs disrupted sleep patterns of males, including a reduction in sleep duration and aggravation of sleep fragmentation. Suppressed activity levels were also found after ZnFe_2_O_4_-NPs exposure. Significant increased expressions of circadian genes (*Clk* and *Cyc*) were detected, alongside elevation of neurotransmitter levels and related gene expressions. **Conclusions**: Overall, ZnFe_2_O_4_-NPs can perturb development process via inducing heat shock and detoxification response, and disrupted rhythmic behaviors may be attributed to elevation of neurotransmitter levels and upregulated gene expressions of circadian genes. Our findings may offer valuable insights for evaluating ecological risks of metal-based nanoparticles and suggest potential applications in developing novel pest management strategies by utilizing insect behavioral and physiological responses to nanomaterials.

## 1. Introduction

With the rapid advancement of nanotechnology in recent decades, utilization of magnetic nanoparticles has been significantly accelerated, leading to their integration into a wide range of fields. Magnetic nanoparticles have been widely applied in various fields, including drug delivery, medical imaging, biosensing, and photocatalysis [1,2,3,4]. Among the magnetic nanoparticles, ferrite particles have garnered particular attention due to their excellent biocompatibility, tunable magnetic properties, and ease of synthesis [5,6]. These characteristics make them ideal candidates for a wide range of applications, including magnetic resonance imaging (MRI), magnetic hyperthermia, and environmental remediation. Ferrite nanoparticles (MFe_2_O_4_) were usually synthesized through metal doping (where M = Fe^2+^, Mg^2+^, Co^2+^, Ni^2+^, Zn^2+^, Mn^2+^, etc.) [7]. In recent years, the zinc ferrite nanoparticles (ZnFe_2_O_4_-NPs) market has seen significant growth, propelled by advancements in nanotechnology and increased adoption in sectors such as healthcare, electronics, and environmental remediation [8]. Ferrite market size was valued at USD 5 billion in 2023 and forecast to reach around USD 6.38 billion by 2030. Zinc ferrite nanoparticles account for about 5% of the ferrite market scale, and it is expected that the annual average production will reach 375 tons by 2030 [9]. Assuming that the release rate in the industrial production process is 0.1–1% (referring to the average release factor of the nanomaterial industry [10]), tons of zinc ferrite nanoparticles may enter the environment every year. Zinc ferrite nanoparticles of around 100 nm can be used not only in photodynamic therapy but also for gas monitoring in the environment [11,12]. Studies have focused on the environmental transport, transformation, and toxicity of engineered nanomaterials such as iron oxides, zinc oxide, silver nanoparticles, and titanium dioxide. These studies have confirmed that these nanomaterials are released into environmental media including soil, water, and air, and trigger related ecological risks [13,14]. Currently, there are no established release standards or regulations for magnetic nanoparticles, raising concerns about their potential adverse effects on living organisms. The potential for magnetic nanoparticle release to environment was expected to increase, while the environmental and ecological risks caused by ZnFe_2_O_4_-NPs release remain unclear.

Recent studies revealed various biological responses to different ferrite nanoparticles. For example, silver ferrite nanoparticles were shown to extend the lifespan of female *Drosophila melanogaster* (*D. melanogaster*) and enhance offspring production [15]. Iron accumulation and toxicity were induced via iron oxide nanoparticles oral intake in *D. melanogaster*, accompanied by weakened female reproductive capacity and delayed developmental transition. A concentration of 1000 mg/kg Fe_3_O_4_-NPs reduced the number of offspring in *Drosophila* [16]. Wing deformities were induced under iron oxide nanoparticle exposure [17], and an 80 μg/mL Fe_3_O_4_ nanocomposite decreased the crawling speed of *Drosophila* larvae and the body weight of adult *Drosophila* [18]. In zebrafish, iron oxide nanoparticles can induce circadian dysregulation, characterized by elevating average activity speed and reducing sleep frequency [19]. Fe_3_O_4_-NPs enhanced photosynthetic pigment content, biomass, and stress resilience in wheat (especially under salinity) and tomato [20]. These findings collectively suggested that upon entering the ecological environment, various ferrite nanomaterials can induce multiple effects on the development, and behavior of both animals and plants. A previous study has found that 200 μM of ZnFe_2_O_4_@poly(tBGE-alt-PA) nanocomposite caused weakened climbing ability, decreased body weight, and broken wing venation in adult flies [21]. Although the applications and releases into the environment are increasingly growing, the mechanisms and bioeffects of ZnFe_2_O_4_-NPs in living organisms remain insufficiently studied.

In this study, *D. melanogaster* was selected as an experimental model not only due to its well-characterized genetic background and short life cycle, but also for its distinct advantages in nanotoxicology research [22]. As a terrestrial organism, it provides relevant insights into nanoparticle exposure through land ecosystems [23]. Its genetic tractability enables precise investigation of nanoparticle uptake and toxicity mechanisms [24]. For example, the dietary intake of AgNPs in the early larval stage led to behavioral abnormalities in *D. melanogaster*, such as poorer crawling and climbing abilities in larvae and adults [25]. Flawed climbing behavior against gravity was seen in ZrO_2_ NP-treated flies [26]. Furthermore, due to sleep architecture of *D. melanogaster* shares fundamental with mammals, the advantages of this model organism have also been essential for understanding the molecular nature of circadian (24 h) rhythms and continue to be valuable in discovering novel regulators of circadian rhythms and sleep. Previous studies have found that metal materials can alter the rhythmic behavior of *D. melanogaster*, affecting activity and sleep. Circadian disruption and sleep disorders are strongly connected to neurodegenerative diseases including Parkinson’s disease, Alzheimer’s disease, and Huntington’s disease as well as others. Metal exposures have been implicated in neurodegenerative diseases, in some cases involving metals that are essential micronutrients but are toxic at high levels of exposure. For example, daily rhythm of activity was disrupted in aged flies under aluminum exposure [27]. These findings collectively suggested that ZnFe_2_O_4_-NPs may probably affect rhythmic behavior of *D. melanogaster*.

In this study, ZnFe_2_O_4_-NPs were hydrothermally synthesized and characterized and oral exposure to ZnFe_2_O_4_-NPs were performed in *D. melanogaster* at concentrations of 0, 200, 400, or 600 μg/mL. Intestinal barrier of parental flies (including male and female) was observed by TEM analysis. Developmental parameters of the offspring were systematically quantified, and rhythmic activity levels and sleep patterns of parent flies were monitored. Simultaneously, mechanisms of ZnFe_2_O_4_-NPs exposure on *D. melanogaster* were elucidated. This integrated study may highlight the urgent need to investigate the mechanisms of environmental release of ferrite nanoparticles and enrich comprehensive understandings about effects of ferrite nanoparticles on development and rhythmic behaviors in insect populations.

## 2. Materials and Methods

### 2.1. Synthesis of ZnFe_2_O_4_-NPs

ZnFe_2_O_4_-NPs were synthesized using the hydrothermal method. A mixture of 1.983 mmol of FeCl_3_·6H_2_O, 0.992 mmol of ZnCl_2_, 8.925 mmol of polyvinylpyrrolidone (PVP, K = 30), and 29.75 mmol of CH_3_COONa was combined in 35 mL of ethylene glycol and stirred magnetically for 4 h to ensure complete dissolution of the components. All these chemicals were obtained from Sinopharm Chemical Reagent Co., Ltd., Shanghai, China. Subsequently, the solution was transferred to a 50 mL Teflon-lined autoclave (Zhengzhou Keda Machinery Company, Zhengzhou, China), heated to 180 °C for 1 h, and then heated to 200 °C for 8 h. The resulting mixture was washed with deionized water and ethanol (Analytical Reagent, Sinopharm Chemical Reagent Co., Ltd., Shanghai, China). Finally, the sample was vacuum-dried at 40 °C for 12 h to obtain the ZnFe_2_O_4_-NPs. The residual pressure during vacuum drying was maintained below 133 Pa.

### 2.2. Characterization of ZnFe_2_O_4_-NPs

The microstructure of the prepared ZnFe_2_O_4_-NPs sample was characterized by using SEM (Verios 460L scanning electron microscope, FEI Corporation, Hillsboro, OR, USA). Crystal structure was analyzed via using X-ray diffractometer analysis (XRD, SmartLab 9KW, Rigaku Corporation, Tokyo, Japan). Chemical bonding state of ZnFe_2_O_4_-NPs was examined by X-ray photoelectron spectroscopy (XPS, Escalab 250Xi system, Thermo Scientific, Waltham, MA, USA).

### 2.3. Exposure to ZnFe_2_O_4_-NPs and Experimental Conditions

In this study, the wild-type *D. melanogaster* (W1118) was utilized (Core Facility of Drosophila Resource and Technology, CEMCS, CAS). The ZnFe_2_O_4_-NPs were thoroughly mixed with the yeast cornmeal standard medium [28] to achieve final concentrations of 200 μg/mL, 400 μg/mL, and 600 μg/mL. These concentrations were determined based on the method described by Chen et al. [29]: using twice the clinical dose of Feridex as the reference. Feridex has a recommended human dose of 0.56 mg Fe/kg body weight [30], and dosage conversion was performed via the body surface area (BSA) ratio between *Drosophila* (8.8 × 10^−6^ m^2^) and humans (1.71 m^2^), yielding an equivalent iron dose of 3.46 × 10^−4^ mg per *Drosophila*. Calculations further showed the total ZnFe_2_O_4_ requirement for 40 parental flies and 100 offspring was 0.528 mg. Correspondingly, the theoretical concentration for a single administration in *Drosophila* was approximately 200 μg/mL, while for daily administration it was approximately 600 μg/mL. Flies of the control groups were maintained on the same standard medium containing inactivated yeast, sucrose, agar, corn meal, and maltose (all the medium chemicals obtained from Sinopharm Chemical Reagent Co. Ltd., Shanghai, China). All the flies were cultivated in an artificial climate incubator (PQX-450A-3HM, Ningbo Laifu Technology, Ningbo, China) with 25 ± 1 °C, 60 ± 2% relative humidity, and 12 h light/dark cycle (lights on at 6:00 AM and off at 6:00 PM).

### 2.4. TEM Analysis of Guts of D. melanogaster

To detect the presence of ZnFe_2_O_4_-NPs in the intestines of the *D. melanogaster*, both male and female parental flies were collected, respectively, and dissected after 72 h exposure to ZnFe_2_O_4_-NPs. The guts were extracted as previously described [31]. Briefly, parental flies were cleaned and dissected in phosphate buffer (PB; 0.1 M, pH 7.4) and fixed for 2 h in a solution containing 4% paraformaldehyde and 1% glutaraldehyde in 0.15 M phosphate buffer (pH 7.4). Gut tissues were post-fixed for 2 h with 1% (*w*/*v*) osmium tetroxide containing 0.8% (*w*/*v*) potassium hexocyanoferrate (prepared in PB), followed by four washes with deionized water and sequential dehydration in acetone. Finally, samples were embedded in Eponate 12^TM^ resin (Ted Pella Inc., Redding, CA, USA) and polymerized at 60 °C for 48 h. Semi-thin sections (1 μm thick) were obtained and stained with 1% (*w*/*v*) aqueous toluidine blue, and then placed on noncoated 200 mesh copper grids, and contrasted with conventional uranyl acetate (30 min) and Reynolds lead citrate (5 min) solutions (All the chemicals mentioned were obtained from Sigma-Aldrich Technology, St. Louis, MO, USA). Sections of samples were observed with a LaB6 Transmission electron microscopy (100 kV TEM, FEI Company, Hillsboro, OR, USA).

### 2.5. Developmental Detection Experiments

For each experimental group (0, 200, 400, or 600 μg/mL ZnFe_2_O_4_-NPs), three replicate culture tubes were prepared, and 20 virgin male–female pairs were collected and placed into each tube. Parental flies were removed after 72 h of oviposition, and cultures were maintained until eclosion of the final offspring (F1 generation). The specific statistical methods about F1 generation were as follows:

Number of pupae: the total number of pupae per tube was counted, with counts commencing upon the emergence of the first pupa and continuing until pupation ceased.

Number of eclosed adults: the cumulative total number of adult *Drosophila* eclosing per tube was recorded from the emergence of the first adult until no further eclosion occurred.

Total offspring count: the sum of the number of pupae and the number of eclosed adults per tube.

Sex ratio: the numbers of male and female *Drosophila* in each tube were recorded separately. The sex ratio was calculated as the number of females divided by the number of males.

Pupation percentages in the first 3 days: the day of the first pupal emergence was designated as day one. The proportion of total pupae formed within the first 3 days was calculated.

Body weight: the weight of each individual eclosed adults (24 h post-eclosion) per tube was determined.

### 2.6. Monitoring of Rhythmic Behavior in Drosophila

Male adults of the F1 generation, collected at 24 h post-eclosion, were used for the behavioral analysis. This experiment followed the established methodology for sleep and rhythm parameter quantification described by Wang et al. [32]. For each concentration group, 96 flies were individually loaded into tubes (inner diameter: 5 mm; length: 65 mm) of the Drosophila Activity Monitor (DAM2) system (TriKinetics, Waltham, MA, USA). Each tube contained a culture medium (5% sucrose/2% agar, chemicals obtained from Sinopharm Chemical Reagent Co., Ltd., Shanghai, China) supplemented with the corresponding concentration of ZnFe_2_O_4_-NPs, and flies were maintained under 12 h:12 h light/dark (LD) conditions throughout the experiment. The DAM2 system recorded the activity of each fly every 5 min for a continuous period of at least 3 days. Sleep was defined as a period of continuous immobility lasting ≥5 min [33]. The differences in the number of activities (activity counts), unit activity capacity (total activity counts/awake time), sleep duration, number of sleep episodes, mean sleep duration per episode, and activity and sleep rhythms were analyzed. Each experiment was repeated three times.

### 2.7. Oxidative Stress Analysis and Neurotransmitter Levels Detection

The F1 generation in each group were collected after 24 h post-eclosion, quick-frozen with liquid nitrogen, and stored at −80 °C for follow-up detection. Reactive oxygen species (ROS) levels, total antioxidant capacity (T-AOC), superoxide dismutase (SOD) activity (WST-1 method), catalase (CAT) activity (ammonium nitrite method), malondialdehyde (MDA) content, gamma aminobutyric acid (GABA) concentration, and acetylcholine (ACh) levels were measured using specific commercial assay kits (Nan Jing Jian Cheng Bio Inst, Nanjing, China), strictly following the manufacturers’ protocols. Integrated optical density (IOD)of Dichlorofluorescein (DCF) were utilized to measure the ROS levels. This method is based on the redox-sensitive fluorescent probe DCFH-DA (2′,7′-Dichlorodihydrofluorescein diacetate), and the detection was performed according to standard protocol of the assay kit. Total protein (TP) concentration was quantified using a Total Protein Assay Kit (Kemas Brilliant Blue method; Nan Jing Jian Cheng Bio Inst, Nanjing, China). All assays were performed in triplicate.

### 2.8. QRT-PCR Detection

The eclosed F1 adults were collected according to the exposure concentration and sex; 30 male and 30 female flies of each concentration were selected for RNA extraction. Total RNA was extracted by TRIzol reagent (Sigma-Aldrich Technology, St. Louis, MO, USA) and reverse transcribed by the Prime Script RT Master Mix Perfect Real Time kit (Takara, Kyoto, Japan), and the Green Premix Ex TaqII kit (Takara, Kyoto, Japan) was used for real-time fluorescence quantitative PCR experiments. The relative expression levels of heat stress family genes (*Hsp26*, *Hsp70*), immune system-related genes (*TotA*, *TotC*), circadian clock genes (*Cyc*, *Clk*), and neurotransmitter-related genes (*Dα1*, *Dβ1*, *ChAT*, *Gat*, *Gad1*) were detected. All samples were tested three times, and the CT values of the target genes were normalized to the CT values of the reference gene *rp49*. The relative quantitative analysis was carried out by the 2^−ΔΔCT^ method [34]. The primers used are shown in Supplementary Table S1.

### 2.9. Statistical Analysis

The experimental data were analyzed using SPSS 20 (IBM Corporation, Armonk, NY, USA) and Origin 2021 (OriginLab Corporation, North Andover, MA, USA). Differences between the control and exposure groups were analyzed by one-way ANOVA, and LSD method was employed to assess significance. Data were presented as mean ± SEM of three independent biological replicates. Statistical results are expressed as the mean ± SEM, *p* < 0.05 represents a statistically significant difference (* *p* < 0.05; ** *p* < 0.01).

## 3. Results

### 3.1. SEM Analysis of Nano ZnFe_2_O_4_ and the TEM Analysis of Intestines of Drosophila melanogaster

**Figure 1:**
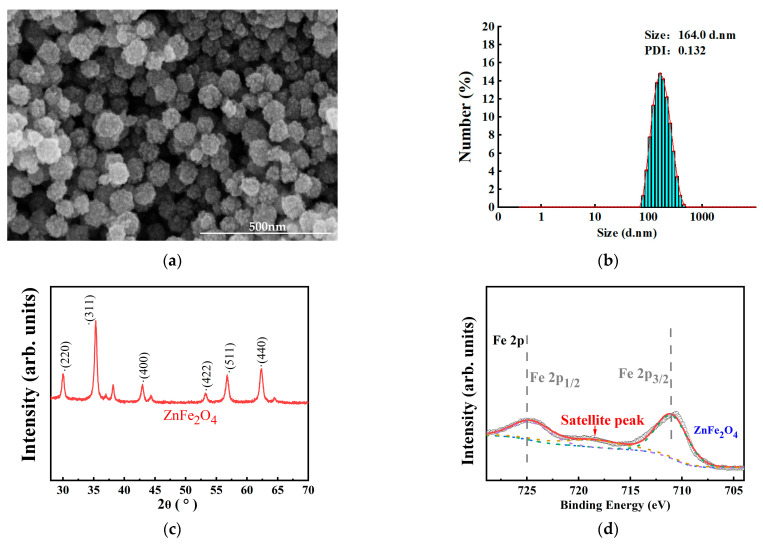

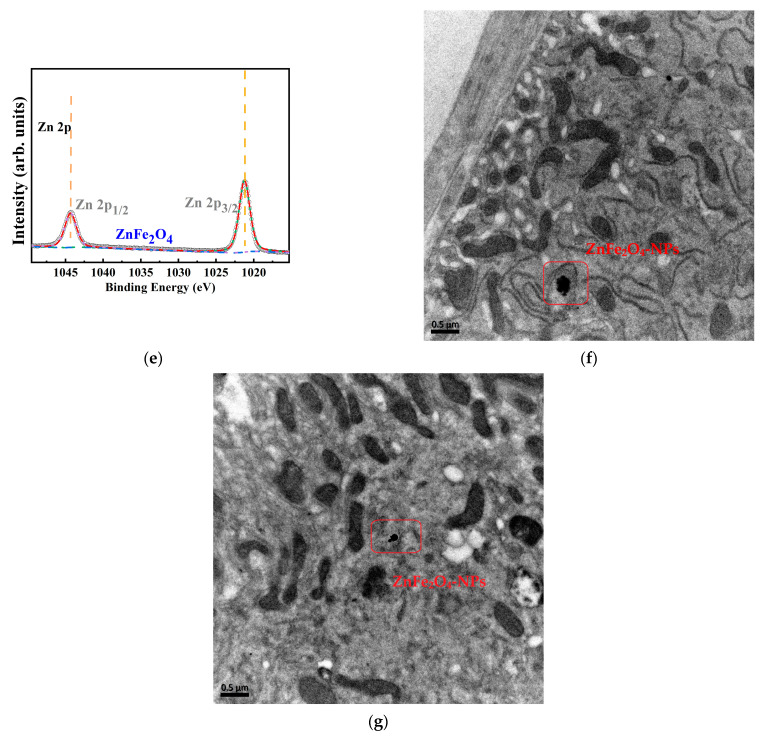
(**a**) The SEM image of ZnFe_2_O_4_-NPs. (**b**) Size distribution of ZnFe_2_O_4_-NPs. (**c**) XRD spectrum of ZnFe_2_O_4_-NPs. (**d**) Fe 2p XPS spectra of ZnFe_2_O_4_-NPs. Scatter points represented the experimental data. The red, blue, green, purple, and brown lines corresponded to the overall sum fit, background, Fe^3+^ 2p_3_/_2_ peak, Fe^3+^ 2p_1_/_2_ peak, and satellite peak. (**e**) Zn 2p XPS spectra of ZnFe_2_O_4_-NPs. Scatter points represented the experimental data. The red, blue, green, and purple lines corresponded to the overall sum fit, background, Zn^2+^ 2p_3_/_2_ peak, and Zn^2+^ 2p_1_/_2_ peak. (**f**) The gut image of the female. (**g**) The gut image of the male.

Morphological analysis by SEM revealed monodisperse spherical particles with an average diameter of approximately 100 nm (Figure 1a). The particle size distribution was further quantified showing a measured value of 164.0 d.nm (Figure 1b). The diffraction peaks observed at 2θ angles of 29.9°, 35.3°, 42.9°, 53.2°, 56.7°, and 62.2° were indexed to the planes (220), (311), (400), (422), (511), and (440), respectively (Figure 1c). The pattern was consistent with the ZnFe_2_O_4_-NPs spinel structure as per PDF card # 79-1150. Additionally, the XPS spectra of Fe(2p) and Zn(2p) were detected. The peaks at 711.7 eV (Fe 2p_3/2_) and 725.4 eV (Fe 2p_1/2_) confirmed Fe^3+^ in the crystal structure, and peaks at 1021.51 eV (Zn2p_3/2_) and 1044.66 eV (Zn 2p_1/2_) indicated the Zn^2+^ state in the spinel structure (Figure 1d,e). TEM analysis demonstrated that internalization of ZnFe_2_O_4_-NPs through the flies’ intestinal barrier was observed (Figure 1f,g).

### 3.2. Effects of ZnFe_2_O_4_-NPs on Development

**Figure 2:**
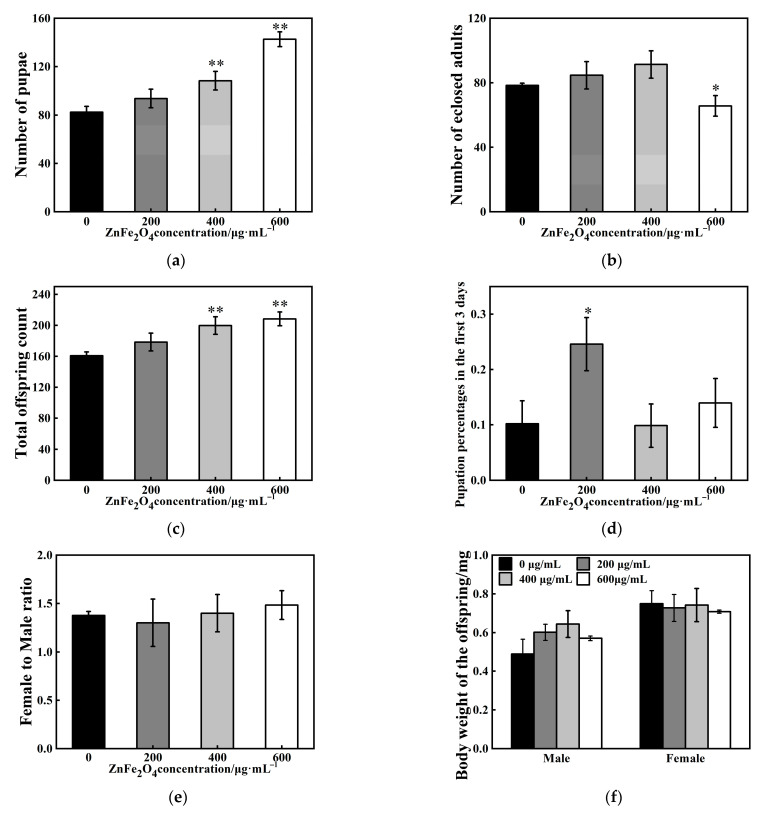
(**a**) Number of pupae. (**b**) Number of eclosed adults. (**c**) Total offspring count. (**d**) Pupation percentages in the first three days (calculated as: number of pupae formed in first 3 days/total pupae count; value of 0.1 indicates 10% of total pupation occurred in the first three days in control group). (**e**) Female to male ratio. (**f**) body weight of the offspring. Values represent mean ± SEM. (* *p* < 0.05; ** *p* < 0.01).

After 3 days of exposure, the parental flies were removed from mating vials and the number of offspring surviving to pupal and adult life-stages was counted. Analysis of total offspring production revealed that ZnFe_2_O_4_-NPs exposure can significantly increase numbers of offspring, with the most pronounced effects observed at higher concentrations (Figure 2a–c). Total pupation counts were elevated by 32% and 73% after 400 μg/mL and 600 μg/mL ZnFe_2_O_4_-NPs exposure, respectively (** *p* < 0.01, Figure 2a). Under 200 μg/mL ZnFe_2_O_4_-NPs exposure, pupation rates within the first 3 days were accelerated by 141% (* *p* < 0.05, Figure 2c). For eclosion development, 600 μg/mL ZnFe_2_O_4_-NPs exposure induced a 16% reduction in the number of eclosed adults (* *p* < 0.05, Figure 2b). No significant differences in female-to-male ratio or body weight of offspring were observed under ZnFe_2_O_4_-NPs exposure (Figure 2e,f).

### 3.3. Effects of ZnFe_2_O_4_-NPs Exposure on Rhythmic Behaviors

**Figure 3:**
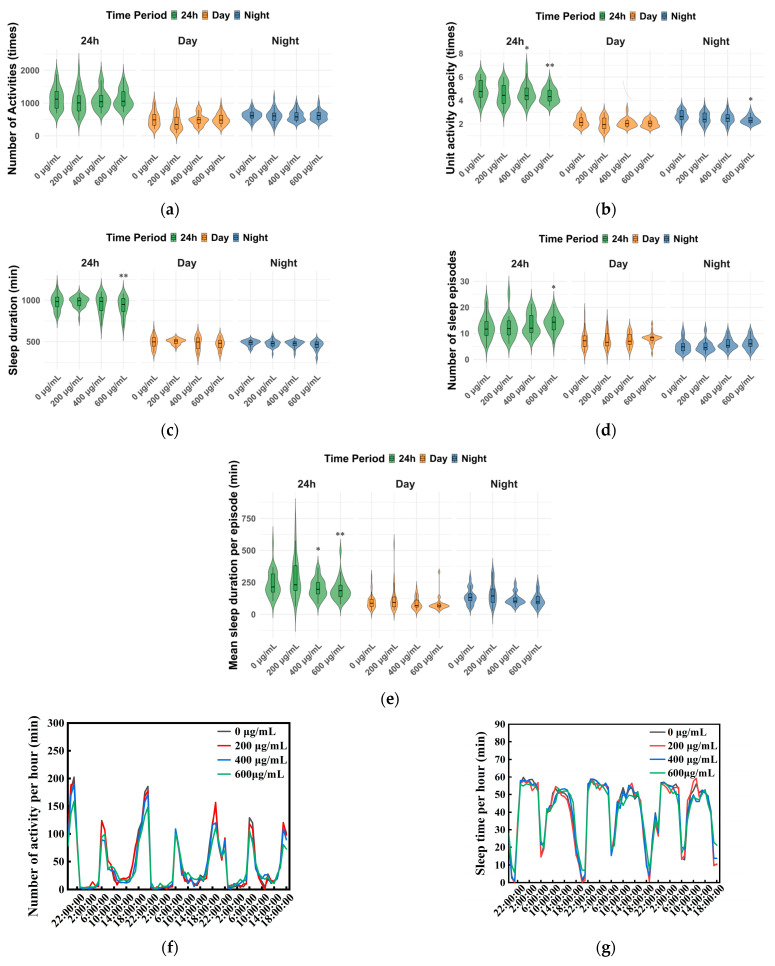
Activity and sleep of male flies under ZnFe_2_O_4_-NPs exposure. (**a**) Number of activities (times). (**b**) Unit activity capacity(times). (**c**) Sleep duration(min). (**d**) Number of sleep episodes. (**e**) Mean sleep duration per episode (min). (**f**) Activity pattern diagram in 24 h. (**g**) Sleep pattern diagram in 24 h. All values are expressed as the means ± SEM. (* *p* < 0.05; ** *p* < 0.01). At least 90 flies were analyzed in each group.

The activity results showed that for male flies after ZnFe_2_O_4_-NPs exposure, decreased unit activity capacity was observed while there were no statistically significant changes in the number of activities (Figure 3a,b). High concentrations of ZnFe_2_O_4_-NPs may induce less unit activity times. The 400 μg/mL ZnFe_2_O_4_-NPs group exhibited 6% decreases in unit activity capacity in 24 h, and the 600 μg/mL ZnFe_2_O_4_-NPs group exhibited 12% and 10% decreases in unit activity capacity in night and 24 h, respectively (* *p* < 0.05, ** *p* < 0.01, Figure 3b). For sleep patterns, decreased sleep duration (5%, ** *p* < 0.01), increased number of sleep episodes (13%, * *p* < 0.01), and decreased mean sleep duration per episode (20%, ** *p* < 0.01) were found under 600 μg/mL ZnFe_2_O_4_-NPs exposure (Figure 3c–e). In addition, no significant changes were observed except a 15% reduction in mean sleep duration per episode under 400 μg/mL ZnFe_2_O_4_-NPs exposure (Figure 3e, * *p* < 0.05). Three-day recording data showed that the exposure groups exhibited similar activity and sleep rhythms as the control groups (0 μg/mL), regardless of concentrations of ZnFe_2_O_4_-NPs (Figure 3f,g), with an activity peak and a sleep trough at ~6:00 and 18:00, the same as the set light/dark cycles (12 h/12 h, light-on time set at 6:00 AM every day, GMT + 6:00).

### 3.4. Oxidative Stress Responses Under ZnFe_2_O_4_-NPs Exposure

**Figure 4:**
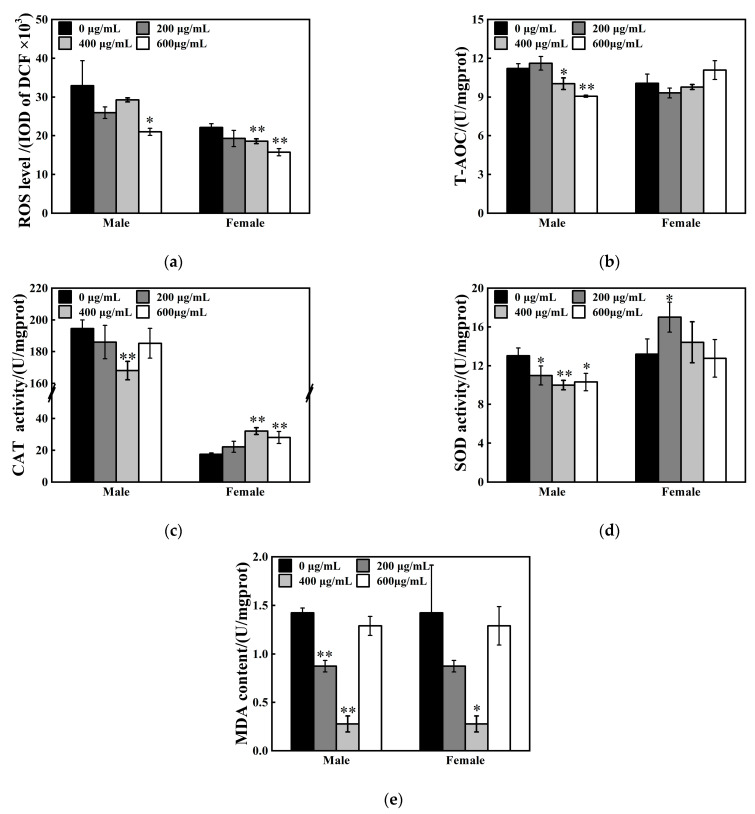
Effects of ZnFe_2_O_4_ on oxidative stress in flies. (**a**) ROS levels (Integrated Optical Density of DCF). (**b**) T-AOC levels. (**c**) CAT activities. (**d**) SOD activities. (**e**) MDA contents. Values represent mean ± SEM. (* *p* < 0.05; ** *p* < 0.01).

The offspring flies were selected and examined after 24 h post-eclosion. The effects of ZnFe_2_O_4_-NPs on oxidative stress responses differ between sexes. ROS levels in vivo decreased by 36% in male flies after 600 μg/mL ZnFe_2_O_4_-NPs exposure, while 16% and 29% reduction in ROS contents in females were found, respectively, after 400 μg/mL and 600 μg/mL ZnFe_2_O_4_-NPs exposure (* *p* < 0.05, ** *p* < 0.01, Figure 4a). For determination of the antioxidant system, T-AOC suppression was found in males flies (11% and 19% under 400 and 600 μg/mL ZnFe_2_O_4_-NPs exposure, respectively), but no significant changes were observed in female flies (Figure 4b). Specifically, ZnFe_2_O_4_-NPs weakened the CAT and SOD enzyme activities of male flies, accompanied with depressed MDA levels (* *p* < 0.05, ** *p* < 0.01, Figure 4c–e). Concurrently, the female flies exhibited upregulated CAT and SOD enzyme activities under ZnFe_2_O_4_-NPs exposure, accompanied with the reduction in MDA contents (* *p* < 0.05, ** *p* < 0.01, Figure 4c–e).

### 3.5. Effects of ZnFe_2_O_4_-NPs on Neurotransmitters Levels

**Figure 5:**
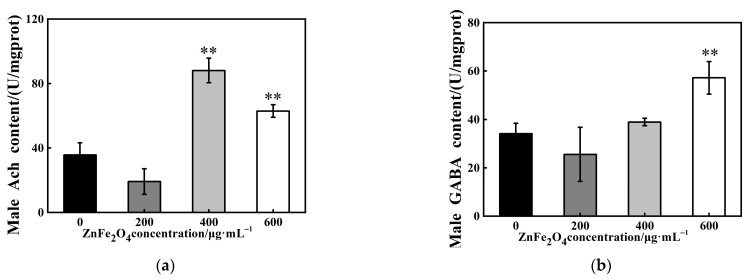
Effects of ZnFe_2_O_4_-NPs on neurotransmitters levels. (**a**) ACh content. (**b**) GABA content. Values represent mean ± SEM. (** *p* < 0.01).

To investigate the mechanisms of rhythmic alterations, we examined the neurotransmitter levels in vivo of F1 male flies after ZnFe_2_O_4_-NPs exposure. The results showed that ACh contents were elevated by 147% and 77% under 400 and 600 μg/mL exposure, respectively (** *p* < 0.01, Figure 5a). In parallel, the 600 μg/mL dose exposure induced a 68% upregulation of GABA (** *p* < 0.01, Figure 5b).

### 3.6. Relative Expression Levels of Target Genes After ZnFe_2_O_4_-NPs Exposure

**Figure 6:**
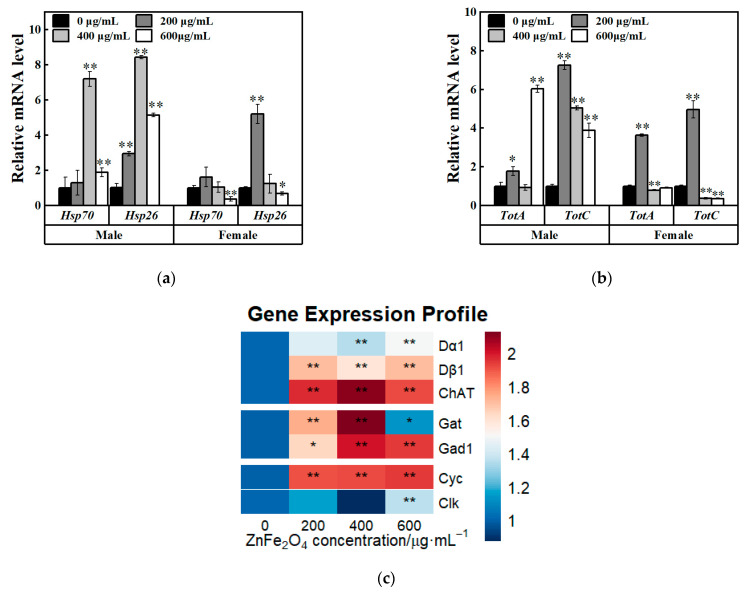
Expression profiles of genes in flies. (**a**) Changes in *Hsp26* and *Hsp70* gene expression in flies. (**b**) Changes in *TotA* and *TotC* gene expression in flies. (**c**) Changes in *Dα1*, *Dβ1*, *ChAT*, *Gat*, *Gad1*, *Cyc*, and *Clk* gene expression in flies. Values represent mean ± SEM. (* *p* < 0.05; ** *p* < 0.01).

To further elucidate the mechanisms of ZnFe_2_O_4_-NPs exposure in insects, relative expression levels of heat shock protein encoding genes (*Hsp70*, *Hsp26*) and Turandot family genes (*TotA*, *TotC*) were investigated. *TotA* is a stress-responsive peptide coordinating trade-offs between immunity and reproduction, while *TotC* can mediate metabolic adaptation to nutrient deprivation in *D*. *melanogaster* [35,36]. In F1 males, significant upregulated relative expression levels of *Hsp70*, *Hsp26*, *TotA* and *TotC* genes were detected (* *p* < 0.05; ** *p* < 0.01, Figure 6a,b). However, downregulated relative expression levels of *Hsp70* and *Hsp26* were found in F1 females (at 600 μg/mL ZnFe_2_O_4_-NPs exposure, * *p* < 0.05; ** *p* < 0.01, Figure 6a), although relative expression levels of *Hsp26* was increased under 200 μg/mL exposure (** *p* < 0.01, Figure 6a). For Turandot family genes, the F1 females exhibited increased relative expressions at 200 μg/mL exposure but decreased gene expressions at 600 μg/mL (** *p* < 0.01, Figure 6b), which indicated that stress adaptation to ZnFe_2_O_4_-NPs exposure differed between sexes.

As shown in Figure 5, acetylcholine (ACh) and GABA levels were elevated in F1 males after ZnFe_2_O_4_-NPs exposure. To further elucidate the mechanism, we analyzed the expression of genes involved in neurotransmitter synthesis and circadian rhythm regulation. Males showed broad upregulations (* *p* < 0.05; ** *p* < 0.01, Figure 6c) of dopamine receptor genes (*Dα1*, *Dβ1*), choline acetyltransferase encoding gene (*ChAT*), glutamic acid decarboxylase (*Gad1*), and GABA transporter encoding gene (*Gat*). Furthermore, compared with the control groups, the exposure groups showed increased relative expressions of transcription factors involved in circadian rhythm regulation, including cycle (*Cyc*) and clock (*Clk*) genes (under 600 μg/mL dose of ZnFe_2_O_4_-NPs, Figure 6c). These upregulations confirm that ZnFe_2_O_4_-NPs exposure can induce neurotransmitter synthesis and further perturb the circadian rhythm of male flies.

## 4. Discussion

Although ZnFe_2_O_4_-NPs have garnered significant interest in biomedicine today, including applications as drug delivery vehicles and in hyperthermia-based tumor therapy, various studies have reported distinct biological interactions and context-dependent toxicity profiles [37,38]. The potential ecological risks associated with ZnFe_2_O_4_-NPs exposure, particularly their effects on insect development and circadian rhythms, remain unclear. In this study, we investigated the developmental and circadian impacts of ZnFe_2_O_4_-NPs exposure using *D. melanogaster* as an in vivo model.

SEM analysis revealed that average diameter of hydrothermally synthesized ZnFe_2_O_4_-NPs in this study was approximately 100 nm, and TEM analysis showed that orally delivered ZnFe_2_O_4_-NPs can be adhered to intestinal microvilli and internalized into gut cytoplasm of both sexes (Figure 1). Nanoscale size enabled penetration through intestinal barriers via mucus permeation and endocytosis, with microvilli as primary attachment sites [39,40,41]. Our results aligned with reports on other metal oxide nanoparticles in *D. melanogaster*. Our results were consistent with previous reports on metal oxide nanoparticle uptake in *D. melanogaster*. For instance, it has been demonstrated that ZnO nanoparticles can adhere to midgut microvilli in *D. melanogaster* [42]. Similarly, studies in Xenopus laevis have shown that ZnO nanoparticles can penetrate the gastrointestinal barrier [43]. The observed microvilli adhesion and cytoplasmic internalization of ZnFe_2_O_4_-NPs mechanistically confirm intestinal barrier penetration, a critical prerequisite for the developmental and circadian disruptions documented in this study.

As shown in Figure 2, exposure to ZnFe_2_O_4_-NPs can increase number of pupae and accelerate pupation progression but reduce number of eclosed adults. Increased pupal counts reflected a genuine enhancement in total progeny rather than developmental delay (Figure 2c). Developmental abnormalities occurred in response to ZnFe_2_O_4_-NPs exposure. Magnetite has a highly reactive surface. It can immobilize metals and other molecules, giving it other functionalities [44]. A previous study revealed that uptake of magnetic (Fe_3_O_4_) nanoparticles can disturbed the oogenesis period in female *Drosophila*, which may be caused by disrupted homeostasis of trace elements such as Fe along the anterior–posterior axis of the fertilized eggs [29]. It was considered an important reason for the abnormal development of *Drosophila*. Moreover, a reduction in larval survival was identified in *Drosophila* associated with the toxic effect of dose-concentration of Chitosan-coated Fe_3_O_4_-NPs, which was attributed to oxidative stress processes previously [45]. In our study, similar oxidative stress responses were confirmed by the characterization of upregulated expressions of *Hsp70* and *Hsp26*, along with inhibited T-AOC after exposure to ZnFe_2_O_4_-NPs (Figure 4b and Figure 6a). However, several reports revealed inconsistent conclusions, for instance, magnetic Fe_3_O_4_ can decrease reproductive capacity and reduced both pupal and adult counts [16]. This discrepancy may arise from distinct properties of different magnetic nanomaterials. Our results demonstrated that oral exposure to ZnFe_2_O_4_-NPs can disrupt the developmental process of *D. melanogaster*, and this may be associated with the oxidative stress response.

Notably, we observed an increase trend of female offspring following ZnFe_2_O_4_-NPs exposure (Figure 2e). This suggested that male individuals may be more susceptible to the developmental toxicity of ZnFe_2_O_4_-NPs, resulting in higher mortality or developmental impairment in males. Such sex-specific sensitivity to environmental stressors has been documented in *Drosophila*; for instance, males exhibit higher mortality under thermal stress or metal exposure due to weaker antioxidant defense and higher metabolic vulnerability [46]. In addition to the abnormal development process, the male flies of F1 generation showed multiple alterations in rhythmic behaviors after intake of ZnFe_2_O_4_-NPs. Activity level weakened and total sleep decreased, and exposure to high doses (400, 600/mL) led to more sleep fragmentations and more weakened unit activity capacity (Figure 3). A previous study verified that in response to reduced nutrient absorption caused by dietary changes or intestinal damage, reduced total sleep was exhibited following exposure to nano-plastics in *D. melanogaster* [47]. TEM analysis in this study revealed that flies can internalize ZnFe_2_O_4_-NPs through the intestinal barrier, and elevated HSPs’ gene expressions suggested that cell damage may exist in ZnFe_2_O_4_-NPs-treated guts. Previous studies have manifested apoptosis after magnetite intake [48]. For instance, the guts of adult fruit flies treated with Fe_2_O_4_@poly(tBGE-alt-PA) nanocomposite were identified with significant nuclear damage compared to the control group [21]. Although fragmentations of sleep were affected by ZnFe_2_O_4_-NPs, we observed no significant changes on sleep and activity rhythmic patterns in *D. melanogaster* (Figure 3f,g). This result suggested that ZnFe_2_O_4_-NPs may act as an adaptable environmental stressor, exerting no disruptive effects on rhythmic periodicity and showing no dose dependence within a specific range. Similarly, a previous study found that following intracerebroventricular injection, no significant disruption of circadian rhythms was observed during the maximum 1-month monitoring period post-administration across the blood–brain barrier [49].

Although ecotoxicity of ZnFe_2_O_4_-NPs is poorly understood, numerous studies using different cell types or animals and different nanomaterials, have suggested that oxidative stress is a major negative effect of nanoparticle use [50,51]. Notably, our results indicated that oxidative stress responses to oral ZnFe_2_O_4_-NPs differed by sex and doses (Figure 4). Males showed significant reductions, whereas females displayed enhanced activities of SOD and CAT after ZnFe_2_O_4_-NPs exposure. Notably, we found that CAT activity in male flies was an order of magnitude higher than in female flies. Similar results were found in Musachio et al.’s measurement of CAT activity in *D. melanogaster*, which indicated that the CAT activity of male flies was generally higher than that of female flies [52]. Sex-specific differences in CAT activity may act as a conserved biological feature. The results of our study showed that there was no order of magnitude difference in ROS level, which may be due to no magnitude differences between males’ T-AOC and females’ T-AOC (Figure 4b). T-AOC integrates the synergistic effects of multiple antioxidant components, which compensates for individual enzyme activity differences and maintains overall redox homeostasis [53]. Even if the activity of CAT enzyme changed dramatically, adjustments of the local antioxidant system and regulation of signaling molecule concentrations may still ensure the maintenance of redox homeostatic balance. In *Drosophila*, sexual differences between expressions of metallothionein have been reported under heavy metal exposures [54]. Metallothionein proteins are usually upregulated in response to diverse stimuli, including essential metals such as zinc and iron to which they specifically bind [55]. Previous reports demonstrated that ZnFe_2_O_4_-NPs may not only agglomerate but also degrade and dissolve to ionic forms for their transport and storage in a non-toxic way in cells [56]. Thus, sexual differences in flies in metallothionein expression may lead to variations in the detoxification capacity of ZnFe_2_O_4_-NPs. In this study, male flies exhibit lower activities of antioxidant enzymes and total antioxidant capacity (T-AOC), indicating that males are more vulnerable than females. Our findings align with recent reports demonstrating sexual dimorphism in *Drosophila*’s response to metal nanoparticle exposure, wherein males exhibit higher susceptibility [57]. Additionally compared to the control groups, both of the ROS and MDA levels exhibited significant reductions in ZnFe_2_O_4_-NPs exposed groups. This found is inconsistent with prior studies, and such effects may be due to the exposed duration and special of ZnFe_2_O_4_-NPs. In this study, we quantified the ROS and MDA levels of the F1 generation flies, which subjected to lifelong ZnFe_2_O_4_-NPs exposure spanning embryonic to adult stages. Compared to short-term exposure, which may trigger a robust antioxidant stress response (such as increased levels of ROS and MDA [58]), long-term exposure of *Drosophila* to ZnFe_2_O_4_-NPs may lead to adaptations through metabolic adaptation or active mechanisms that clear ROS and MDA. Previous studies [59,60] have confirmed that *Drosophila* can elicit ROS and MDA reduction in adaption to long-term or chronic exposure to different stress factors. Our results revealed concentration-dependent effects on MDA levels in response to ZnFe_2_O_4_-NPs. MDA was significantly elevated under 400 μg/mL exposure, while no significant difference was identified at 600 μg/mL compared to the control, which may be caused by extensive aggregation of ZnFe_2_O_4_-NPs at this concentration. As shown in Bélteky’s study [61], aggregated AgNPs failed to effectively penetrate the insect intestinal cell membrane and thus led to a significant reduction in intracellular accumulation. High concentrations of AgNPs form large aggregates on the surface of intestinal villi and inside cells, which may further correlate with a decline in MDA levels [62].

Moreover, significant increase in expression levels of heat shock protein genes (Figure 6a, *Hsp26*, *Hsp70*) and Turandot protein genes (Figure 6b, *TotA*, *TotC*) in both sexes were identified *in this study*, which further suggested that oxidative stress responses were induced by ZnFe_2_O_4_-NPs. In *D. melanogaster*, silver nanoparticles can activate heat shock protein 70, oxidative stress and apoptosis [63]. Oxidative stress induced by silica nanoparticles might have been involved in proinflammatory responses [64]. Turandot genes, like HSPs, have been characterized to respond to a variety of stress types such as heat stress, cold stress, irradiation, infection, dehydration, oxidative agents, and mechanical stress [65]. Thus, flies may activate Turandot genes expressions in respond to ZnFe_2_O_4_-NPs exposure that can be considered as an adaptable stress factor. In our study, we observed a distinct concentration-dependent pattern for HSPs. High nanoparticle concentration exposure may inhibit HSPs’ production. Upregulated expression levels of HSPs were detected at 400 μg/mL in male flies and 200 μg/mL in female flies, while expression levels were declined significantly under 600 μg/mL exposure (Figure 6a). This decline may be attributed to the aggregation of ZnFe_2_O_4_-NPs, which can reduce the cellular bioavailability of the nanoparticles. Similar results were founded that high concentrations of AgNPs tend to aggregate and decreased the uptake efficiency in insect gut cells [61]. For ZnFe_2_O_4_-NPs, aggregation may occur at 600 μg/mL concentration and can induce reduction in bioavailability, ultimately leading to lower heat shock protein production. TotA’s mRNA levels in females also indicated that aggregated ZnFe_2_O_4_-NPs nanoparticles may occur under high concentrations. A concentration of 200 μg/mL is the maximum for female flies, and high concentrations reduced the production compared to the maximum, even inhibit it compared to the control. However, the expression level of TotA in male flies under 600 μg/mL was highly upregulated, which was different with the females’ pattern. This may due to sex-specific differences in *Drosophila* in response to different stressors [66].

This study investigated the impact of ZnFe_2_O_4_-NPs on *Drosophila* activity and sleep, revealing significant effects: weakened activity capacity, shortened total sleep time and increased sleep fragmentations (Figure 3). These findings align with previous research demonstrating that exposure to strontium ferrite and magnetic iron oxide nanoparticles similarly reduces climbing ability in fruit flies [67]. Simultaneously, ZnFe_2_O_4_-NPs treatment increased the expression of core clock genes *Cyc* or *Clk* in *Drosophila* (Figure 6), potentially disturbing circadian neurons governing sleep–wake transitions and contributing to unstable sleep and frequent awakenings [68,69]. Altered reactive oxygen species (ROS) levels, which has been confirmed as a consequence of ZnFe_2_O_4_-NPs exposure, can disrupt the circadian clock, which functions to minimize oxidative damage by regulating rhythmic processes [70]. Concurrently, we found elevated levels of the neurotransmitters ACh and GABA in *Drosophila* (Figure 5), and gene expression analysis revealed upregulations of dopamine receptors genes (*Dα1*, *Dβ1*), Choline acetyltransferase (*ChAT*), Glutamic acid decarboxylase (*Gad1*), and GABA transporter (*Gat*) (Figure 6). These findings indicated the enhanced synthesis of neurotransmitters. Elevated ACh levels promote wakefulness [71,72], which can increase sleep fragmentation and reduce stability [73], correlating with the observed increased sleep episodes of flies [74]. GABA is primarily an inhibitory neurotransmitter promoting sleep [75], its increased levels, potentially exacerbated by nanomaterial exposure, may excessively suppress neuronal activity and reduce locomotion [76]. Critically, ZnFe_2_O_4_-NPs directly drive neurotransmitter alterations through transcriptional activation. Notably, GABA functions dually as both a neurotransmitter and an endogenous antioxidant, and its increase likely contributes to ROS suppression. Therefore, ZnFe_2_O_4_-NPs likely can impair sleep quality and activity capacity in *Drosophila* through combined disruptions of the circadian clock and dysregulation of neurotransmitter systems.

## 5. Conclusions

In this study, the effects of ZnFe_2_O_4_-NPs on *D. melanogaster* were investigated. The results indicated that ZnFe_2_O_4_-NPs can enter the gut tissue and act as an external stimulus to upregulate the gene expression levels of HSPs and Turandot proteins, leading to alterations in numbers of pupae and eclosed adults and abnormality of development process. Concurrently, exposure to ZnFe_2_O_4_-NPs can affect the rhythmic behaviors of *D. melanogaster*, leading to weakened activity capacity, significant reduction in total sleep time and increased sleep fragmentations in males. Such effects may be due to the induced oxidative stress responses, increased neurotransmitter levels and upregulated expression levels of circadian genes via exposure of ZnFe_2_O_4_-NPs. Our study further elucidated the response of insect like organisms to ZnFe_2_O_4_-NPs, providing a basis for the rational evaluation of the ecological safety of ZnFe_2_O_4_-NPs. Our findings may provide insights for evaluating ecological risks of metal-based NPs and suggest applications in developing pest management strategies via insect responses to nanomaterials.

## Data Availability

The original contributions presented in this study are included in the article. Further inquiries can be directed to the corresponding author.

## Supplementary Materials

The following supporting information can be downloaded at: https://www.mdpi.com/article/10.3390/toxics13090779/s1. The primer sequences mentioned in this study were supplied in Supplementary Table S1.
