# Supplementary material for: Effects of ZnFe2O4 Nanoparticles on Development and Rhythmic Behavior of Drosophila melanogaster
Source: Toxics. 2025 Sep 14;13(9):779. doi: 10.3390/toxics13090779 (PMC12474399; doi:10.3390/toxics13090779)
Supplement: Supplementary file 1 [file toxics-13-00779-s001.zip › toxics-3851688-supplementary.pdf]

Table S1. Primer sequences.

Gene name	Forward primer (5'-3')	Reverse primer (3'-5')
Hsp26	CGACTCCATCTTGGTCGAGG	TGTAGCCATCGGGAACCTTG
Hsp70	GAACTCACACACAATGCCTGC	TCCGAGTCTGTGAAAGCCA
TotA	TGCTGCTGATTAGTCCTCTATG	GATGTCAAGTAGTTCCTGGGTG
TotC	GATCAATCGAACCCAAGAGC	TTGTGCCACTGGAGATAGGA
Sod1	TCAACATCACCGACTCCAAG	TCAGCTCGTGTCCACCCT
Cat	TTCCAGAGCGTGTCGTGC	CAGACGCCATCCTCAGTGTAG
Cyt-c-p	CAAGAAGTACATCCCCGGCA	ACTTGGTCGCCGACTTCAGG
Dα1	TATGACAACCAATGTCTGGG	CATATATGCTCGGAGGGAAC
Dβ1	TGGTCAGGATAGAACGAGAT	AATTAAAAGTCCCAGGCCAT
ChAT	GCGAAAGGTCAAGTTTAGCA	CTTCACCATCACCTCAGTC
Gat	TAATAGGATTTATGGCTCACG	CTGCCGATGGATAGACAAG
Gad1	CACTGTGCTGGGTGCCT	CTGGGATGACGATGCTTAC
Cyc	GCGAAAGCTGGACAAACTGACT	CCGTTGAATGGATGTAAGCTGC
Clk	ACGAGAGCGACGACAAGGATG	GGCTATCGTGGACTTCAGGACC
RP49	AGCATACAGGCCCAAGATCG	GTTGTCGATACCCTTGGGCT
